# *FMR1* mRNA from full mutation alleles is associated with ABC-C_FX_ scores in males with fragile X syndrome

**DOI:** 10.1038/s41598-020-68465-6

**Published:** 2020-07-16

**Authors:** Emma K. Baker, Marta Arpone, Claudine Kraan, Minh Bui, Carolyn Rogers, Michael Field, Lesley Bretherton, Ling Ling, Alexandra Ure, Jonathan Cohen, Matthew F. Hunter, Lorena Santa María, Victor Faundes, Bianca Curotto, Paulina Morales, Cesar Trigo, Isabel Salas, Angelica Alliende, David J. Amor, David E. Godler

**Affiliations:** 1 Diagnosis and Development, Murdoch Children’s Research Institute, Royal Children’s Hospital, 50 Flemington Road, Parkville, VIC 3052 Australia; 2 Department of Paediatrics, Faculty of Medicine, Dentistry and Health Sciences, University of Melbourne, Parkville, VIC Australia; 3 School of Psychology and Public Health, La Trobe University, Bundoora, VIC Australia; 4 Brain and Mind, Murdoch Children’s Research Institute, Royal Children’s Hospital, Melbourne, VIC Australia; 5 Centre for Epidemiology and Biostatistics, Melbourne School of Population and Global Health, University of Melbourne, Carlton, VIC Australia; 6 Genetics of Learning Disability Service, Hunter Genetics, Hunter New England Health, Waratah, NSW Australia; 7 Neurodisability and Rehabilitation, Murdoch Children’s Research Institute, Royal Children’s Hospital, Melbourne, VIC Australia; 8 Royal Children’s Hospital, Melbourne, VIC Australia; 9 Department of Pediatrics, Monash University, Clayton, VIC Australia; 10 Fragile X Alliance Inc, North Caulfield, VIC, Australia; 11 Centre for Developmental Disability Health Victoria, Monash University, Clayton, VIC Australia; 12 Monash Genetics, Monash Health, Clayton, VIC Australia; 13 Laboratory of Molecular Cytogenetics, Department of Genetics and Metabolic Diseases, Institute of Nutrition and Food Technology (INTA), University of Chile, Santiago, Chile

**Keywords:** Neurodevelopmental disorders, Gene expression

## Abstract

Fragile X syndrome (FXS) is caused by a hypermethylated full mutation (FM) expansion with ≥ 200 CGG repeats, and a decrease in *FMR1* mRNA and its protein. However, incomplete silencing from FM alleles has been associated with more severe autism features in FXS males. This study compared scores on the Aberrant Behavior Checklist-Community-FXS version (ABC-C_FX_) in 62 males affected with FXS (3 to 32 years) stratified based on presence or absence of mosaicism and/or *FMR1* mRNA silencing. Associations between ABC-C_FX_ subscales and *FMR1* mRNA levels, assessed using real-time PCR relative standard curve method, were also examined. The FXS group mosaic for premutation (PM: 55–199 CGGs) and FM alleles had lower irritability (p = 0.014) and inappropriate speech (p < 0.001) scores compared to males with only FM alleles and complete loss of *FMR1* mRNA. The PM/FM mosaic group also showed lower inappropriate speech scores compared to the incomplete silencing (p = 0.002) group. Increased *FMR1* mRNA levels were associated with greater irritability (p < 0.001), and lower health-related quality of life scores (p = 0.004), but only in the incomplete silencing FM-only group. The findings suggest that stratification based on CGG sizing and *FMR1* mRNA levels may be warranted in future research and clinical trials utilising ABC-C_FX_ subscales as outcome measures.

## Introduction

Fragile X syndrome (FXS) is caused by a large trinucleotide CGG expansion (≥ 200 repeats), termed full mutation (FM), in the promoter region of the Fragile X Mental Retardation 1 (*FMR1*) gene^1^. FM alleles are associated with DNA methylation (DNAm) changes to the *FMR1* promoter, resulting in decreased transcription^2^ and little to no production of the Fragile X Mental Retardation Protein (FMRP). FXS is the leading single gene cause of intellectual disability (ID), with autism spectrum disorder (ASD) features also commonly occurring. Smaller premutation (PM) expansions (CGG: 55–199 repeats) typically have an unmethylated *FMR1* promoter, but elevated mRNA levels^3,4^. This elevated *FMR1* mRNA has been associated with “RNA gain of function” toxicity in some PM carriers and has been implicated in late onset disorders such as Fragile X-associated Tremor/Ataxia Syndrome (FXTAS)^5^. Rare unmethylated FM (UFM) alleles in adult individuals have been associated with neurodegeneration observed as FXTAS, hypothesised to be related to “RNA gain of function” toxicity originating from UFM alleles^6–8^.

There is now evidence from independent studies^9–11^, demonstrating that in the majority of FM males, *FMR1* mRNA is not completely silenced (i.e., there is still residual transcription, even in the presence of FM alleles). These studies have reported that between 44 and 60% of FXS males express *FMR1* mRNA^4,10,11^, with this incomplete silencing more recently associated with elevated ASD features in FM-only males, but not intellectual functioning deficits^11^. This suggests that two reciprocal mechanisms, RNA toxicity and FMRP deficiency, may contribute to overlapping aspects of FXS, specifically ID and ASD features. This theory is supported by research demonstrating significant associations between *FMR1* methylation and FMRP, and intellectual functioning parameters^12–14^. However, relationships between *FMR1* molecular variables and maladaptive behaviours assessed using the Aberrant Behavior Checklist-Community fragile X version (ABC-C_FX_)^15^—a tool often used as an outcome measure in clinical trials, have not been thoroughly investigated.

This study aimed to determine if maladaptive behaviours are increased, as measured by the ABC-C_FX,_ in males affected with FXS with complete and incomplete silencing of FM alleles, as compared to males mosaic for PM and FM alleles. The study also explored relationships between the levels of *FMR1* mRNA (if not completely silenced) in Peripheral Blood Mononuclear Cells (PBMCs) and each of the ABC-C_FX_ subscale scores, total score, and the utility index. Based on our previous study^11^, it was hypothesised that FM-only males with incomplete silencing of FM alleles would have elevated scores on the ABC-C_FX_ compared to males with complete silencing of FM alleles.

## Methods

### Participants

Participants were Australian and Chilean males with FXS aged between 3 and 32 years old recruited into previous studies^11, 14^. All participants had undergone fragile X genetic testing prior to recruitment using CGG PCR sizing and Southern blot analysis. Briefly, routine FXS testing involved first-line PCR-based assessment of CGG repeat size (± 1 CGG) with the upper limit of detection being 330 CGG and 170 CGG repeats for the Chilean^16^ and Australian^17^ samples, respectively. DNA samples from all males who showed a CGG size in the PM range or failed to show a PCR product, were reflexed for methylation sensitive Southern to confirm molecular diagnosis of FXS^18,19^. Exclusionary criteria for the study included any other genetic conditions of known clinical significance, if they had any significant medical conditions (e.g., stroke, head trauma), and/or if they had inadequately controlled seizures.

### Sample processing

PBMCs were isolated from 5 ml of blood collected in EDTA tubes, using Ficoll gradient separation. RNA was then extracted from the isolated PBMCs using RNeasy kit as per manufacturer’s instructions (Qiagen, Global) for gene expression analyses.

### *FMR1* mRNA analysis

RNA (10 nanograms per sample) was reverse transcribed using the High Capacity cDNA Reverse Transcription kit, as per manufacturer’s instructions (Thermo Fisher scientific, Global). The ViiaTM 7 system (Thermo Fisher Scientific, Global) was then used to analyse gene expression using the relative standard curve method^20^. Specifically, a series of doubling dilutions of RNA (160–0.5 ng/µl) of a selected PBMC sample was performed for *FMR1* 5′ and 3′ mRNA assays and the two internal control genes (*EIF4A2* and *SDHA*), previously shown to be the optimal control gene combination of gene expression normalization in *FMR1* related disorders^21^. Previously published sequences were used for real-time PCR primers and probes for: *FMR1* 5′ assay targeting exon3/4 junction^22^; and *FMR1* 3′ assay targeting the exon13/exon14 junction^23^. *FMR1* primers and probes were used at 18 µM and 2 µM, respectively. *EIF4A2* and *SDHA* primer/probe mixes were obtained from PrimerDesign (PerfectProbe gePP-12-hu kit) and used at concentration of 2 µM. Each sample was assayed in triplicate in a total volume of 10ul master-mix reaction. The *FMR1* targeting reaction consisted of 5 µl of 2× SensiFAST Probe Low-rox Mix from SensiFAST™ Probe Low-ROX Kit (Bioline, Australia), 2.5 µl of RNase free water, 0.5 μl of TaqMan probe and 0.5 2 µM l forward and 0.5 2 µM l reverse primers, and 1 µl of the reverse transcription (cDNA) reaction. While *EIF4A2* and *SDHA* qPCR reaction is made of 5 µl of 2× SensiFAST Probe Low-rox Mix from SensiFAST™ Probe Low-ROX Kit (Bioline, Australia), 3 µl of RNase free water, 1 μl Primer/Probe mix, and 1 µl of cDNA reaction. The annealing temperature for thermal cycling protocol was 60 °C for 40 cycles. Samples were quantified in arbitrary units (au) in relation to the standard curves performed on each plate with mean of three technical replicates being the representative value of relative *FMR1* mRNA normalized by average internal control gene levels for each sample analysed.

### Intellectual functioning

Intellectual functioning was determined using an age- and language- (English or Spanish) appropriate Wechsler intelligence scale. Specifically, children aged 3 years to 6 years 11 months completed the Wechsler Preschool and Primary Scale of Intelligence-3rd Edition (WPPSI-III) Australian^24^ and Mexican^25^ Editions. Australian children aged 7 years to 16 years, 11 months completed the Wechsler Intelligence Scale for Children-4th edition (WISC-IV) Australian standardised edition^26^ and Chilean children of the same age range completed the Wechsler Intelligence Scale for Children-3rd edition (WISC-III) Chilean edition^27^. Participants aged 17+ years completed the Wechsler Adult Intelligence Scale-4th (WAIS-IV) Australian and New Zealand^28^ and Chilean^29^ editions.

### Maladaptive behaviours

Maladaptive behaviours were assessed using the ABC-C_FX_^15^. The ABC-C_FX_ has six subscales which measure irritability, lethargy, stereotypy, hyperactivity, inappropriate speech, social avoidance. An overall total score can also be calculated by summing up the scores obtained in each subscale. In the current sample these subscales demonstrated good internal consistency (Cronbach’s α = 0.82–0.94). The utility index (UI) to determine FXS health-related quality of life was also used^30^. Higher scores on the subscales indicate greater impairment, while lower scores on the UI indicate poorer health-related quality of life.

### Procedure

Participants attended an appointment for assessment and venous blood collection. Parents/caregivers completed the ABC-C at the time of assessment with the assistance of a research team member, if required. All procedures were approved by The Royal Children’s Hospital and INTA Human Research Ethics Committees (HREC #33066 and #15, respectively). All procedures were performed in accordance with these ethics approvals. All parents/caregivers provided written informed consent and those participants deemed cognitively able also provided written informed consent.

### Statistical analysis

Distribution for each demographic variable and maladaptive behaviours were normally distributed in each of three male groups, namely FM-only with complete silencing, FM-only with incomplete silencing and PM/FM mosaic, and therefore the mean and standard deviation were presented as summary statistics, and analysis of variance was used to compare the difference. Whereas for *FMR1* mRNA, the distribution was not normally distributed in each group, the non-parametric Kruskal–Wallis test was used to compare the difference between the three subgroups or Mann–Whitney U test for pairwise comparisons. For binary data (seizures, country and medication used) the percentage was given, and Fisher’s exact test was used for comparisons. While for maladaptive behaviours, analysis of covariance was used for comparisons, adjusting for age. Robust regression with robust standard error was used to assess the relationship between each maladaptive behaviour score (outcome) and *FMR1* mRNA (predictor) for the combined data, adjusted for age and allelic class, and separately for FM-only with incomplete silencing and PM/FM mosaic, adjusted for age only. The difference in the relationship between these two groups was tested using an interaction term between subgroup (binary) and *FMR1* mRNA levels. Significance of the interaction term indicated that the relationship was different between two subgroups. The Bonferroni correction method was used to correct for multiple testing. All analyses were conducted using Stata (https://www.stata.com).

## Results

The cohort of 62 males with FXS were split into three classifications: FM-only with complete *FMR1* silencing, FM-only with incomplete *FMR1* silencing, and PM/FM mosaics. These three groups did not significantly differ on age, intellectual functioning, and medication use (Table 1). One FM-only male displayed extremely elevated ABC-C_FX_ scores that were atypical in comparison to the remainder of the group. This individual was excluded from the analyses so as not to affect the generalisability of the results. Key demographic and clinical information for those included in the analyses are in Table 1.

**Table 1:** Demographic and clinical information.

	FM-only with complete silencing		FM-only with incomplete silencing		PM/FM mosaic		*p*^1^	*p*^2^	*p*^3^	*p*^4^
	*n*	*M* ± *SD*	*n*	*M* ± *SD*	*n*	*M* ± *SD*				
Age	19	14.6 ± 9.48	29	13.3 ± 7.29	14	16.8 ± 14.8	0.562	0.679	0.519	0.285
FSIQ	9	48.6 ± 13.4	18	43.9 ± 5.74	9	52.6 ± 9.06	0.067	0.211	0.351	**0.024**
ADOS CSS	17	6.06 ± 2.38	26	7.50 ± 1.45	12	6.67 ± 1.83	**0.050**	0.016*	0.390	0.205
Seizures (% lifetime presence)	19	10.5%	29	17.2%	12	0.0%	0.383	0.687	0.510	0.298
Country (% Australian)	19	15.8%	29	37.9%	14	42.9%	0.178	0.119	0.122	0.999
Medicated (% ≥ 1 medication)	19	52.6%	29	58.6%	14	28.6%	0.190	0.770	0.286	0.104
Stimulant	19	31.6%	29	27.6%	14	14.3%	0.566	0.999	0.416	0.456
SSRI	19	21.1%	29	27.6%	14	7.1%	0.318	0.739	0.366	0.231
Antipsychotic	19	5.3%	29	17.2%	14	7.1%	0.512	0.381	0.999	0.645
AAA	19	5.3%	29	0.0%	14	7.1%	0.279	0.396	0.999	0.326
SNRI	19	0.0%	29	0.0%	14	7.1%	0.226	NA	0.424	0.326
Benzodiazepine	19	0.0%	29	0.0%	14	7.1%	0.226	NA	0.424	0.326

P-values highlighted in bold were significant prior to adjustment for multiple comparisons.

*FM* full mutation, *PM* premutation, *M* mean, *SD* standard deviation, *NA* not applicable, *FSIQ* full scale IQ, *ADOS CSS* autism diagnostic observation schedule calibrated severity score, *SSRI* selective serotonin reuptake inhibitor, *AAA* alpha adrenergic agonist, *SNRI* selective norepinephrine reuptake inhibitor.

p-value (*p*) for comparing the means between ^1^three groups; ^2^FM-only with complete silencing and FM-only with incomplete silencing; ^3^FM-only with compete silencing and PM/FM Mosaic; ^4^FM-only with incomplete silencing and PM/FM mosaic.

*P-value remained < 0.05 after adjusting for multiple comparison using Bonferroni correction method.

### *Comparison between the three FXS groups on FMR1 mRNA and ABC-C*_*FX*_* scores*

**Figure 1:**
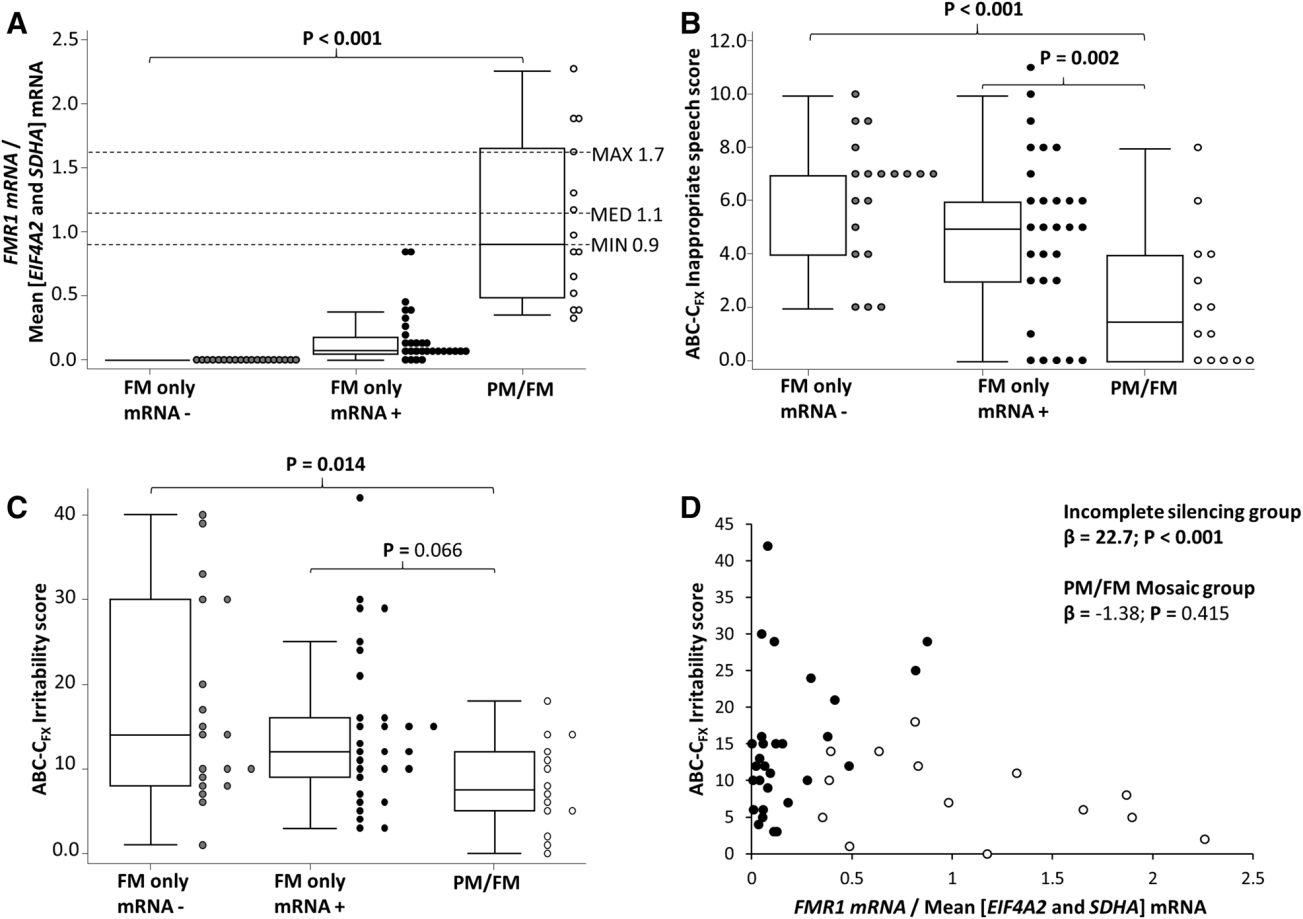
Intergroup comparisons and relationship between *FMR1* mRNA levels in PBMCs and ABC-C_FX_ sub-scales in FM only and PM/FM mosaic males. Intergroup comparisons for (**A**) normalized *FMR1* mRNA levels; Note: Broken parallel lines represent minimum (MIN), maximum (MAX) and median (MED) mRNA values from TD controls (n = 14), from a previous study^11^. Intergroup comparisons for (**B**) ABC-C_FX_ Inappropriate speech scores; and (**C**) ABC-C_FX_ Irritability scores between FM only and PM/FM mosaic males. (**D**) Relationship between normalized *FMR1* mRNA levels and ABC-C_FX_ Irritability score, with open and closed circles representing PM/FM mosaic and FM only incomplete silencing groups, respectively.

All three groups significantly differed from each other on *FMR1* mRNA levels (Fig. 1A). The two FM-only groups did not significantly differ on any of the ABC-C_FX_ scores, though there was a trend towards more stereotyped behaviours in the complete silencing group (Table 2; Fig. 1B,C). The PM/FM mosaic group had significantly lower scores on the Irritability domain (Table 2; Fig. 1C) and ABC-C_FX_ total score, as well as a significantly higher UI compared to the FM-only group with complete silencing (Table 2). Both FM-only groups had significantly elevated scores on the Inappropriate Speech domain compared to the PM/FM mosaic group (Table 2; Fig. 1B).

**Table 2:** Comparison between complete and incomplete silencing *FMR1 m*RNA in FM-only males and PM/FM mosaics on maladaptive behaviours.

	FM-only with complete silencing (N = 19)		FM-only with incomplete silencing (N = 29)		PM/FM mosaic (N = 14)		*p*^1^	*p*^2^	*p*^3^	*p*^4^
	*M*	SD	*M*	SD	*M*	*SD*				
Irritability	16.9	11.8	14.7	9.36	8.07	5.36	**0.045**	0.350	0.014*	0.066
Lethargy	5.79	3.78	6.93	4.64	4.93	3.75	0.472	0.414	0.700	0.252
Stereotypy	6.37	5.14	4.28	3.45	3.93	4.20	0.132	0.055	0.139	0.884
Hyperactivity	13.0	8.37	10.2	7.04	7.29	5.90	0.082	0.094	**0.034**	0.424
Inappropriate speech	6.11	2.38	4.76	3.09	2.21	2.52	< 0.001*	0.119	< 0.001*	0.002*
Social avoidance	3.95	3.19	3.69	2.02	2.93	3.32	0.595	0.732	0.318	0.442
ABC total	52.1	27.4	44.5	18.6	29.4	19.8	**0.020**	0.158	0.005*	0.074
ABC UI	0.57	0.19	0.60	0.14	0.72	0.13	**0.030**	0.415	0.010*	**0.037**

P-values highlighted in bold were significant prior to adjustment for multiple comparisons.

Comparisons were conducted using analysis of covariance, adjusted for age; *FM* full mutation, *PM* premutation, *M* mean, *SD* standard deviation, *ABC* aberrant behavior checklist, *UI* utility index.

p-value (*p*) for comparing the means between ^1^three groups; ^2^FM-only with complete silencing and FM-only with incomplete silencing; ^3^FM-only with compete silencing and PM/FM Mosaic; ^4^FM-only with incomplete silencing and PM/FM mosaic.

*p-value remained < 0.05 after adjusting for multiple comparison of the three pairwise tests using Bonferroni correction method.

### *Relationships between FMR1 mRNA and ABC-C*_*FX*_* scores*

When the incomplete mRNA silencing and PM/FM mosaic groups were combined, no significant associations between *FMR1* mRNA and ABC-C_FX_ scores were observed (Table 3; Fig. 1D). When examining the specific allelic sub-groups*, FMR1* mRNA was not significantly associated with any of the ABC-C_FX_ scores in the PM/FM mosaic male group (Table 3). In the incomplete silencing FM-only group, after Bonferroni correction, *FMR1* mRNA was significantly associated with scores on the Irritability subscale, ABC-C_FX_ Total and ABC-C_FX_ UI (Table 3; Fig. 1D), and these relationships were all significantly different from that of the PM/FM mosaic group (p < 0.001, 0.005 and 0.001, respectively; Table 3).

**Table 3:** Relationship between each maladaptive behaviours (outcome) and *FMR1* mRNA (predictor) by allelic sub-group.

	All (n = 43)			FM-only with incomplete silencing (n = 29)			PM/FM mosaic (n = 14)		
	*β*	s.e	*p*	*β*	s.e	*p*	*Β*	s.e	*p*
Irritability	3.78	2.95	0.199	22.7	3.17	< 0.001*	− 1.38	1.69	0.415
Lethargy	0.28	0.80	0.723	1.44	2.08	0.489	− 0.03	0.86	0.969
Stereotypy	− 1.17	1.80	0.517	− 4.27	1.66	**0.010**	0.01	3.97	0.997
Hyperactivity	− 0.15	1.80	0.932	2.58	5.96	0.665	− 1.12	1.24	0.367
Inappropriate speech	− 1.69	1.57	0.282	0.66	4.59	0.886	− 1.41	1.19	0.239
Social avoidance	0.09	1.15	0.935	1.52	1.61	0.343	− 0.45	1.39	0.744
ABC total	1.93	5.72	0.736	22.4	7.11	0.002*	− 3.66	5.96	0.539
ABC UI	− 0.03	0.05	0.560	− 0.22	0.08	0.004*	0.01	0.05	0.870

P-values highlighted in bold were significant prior to adjustment for multiple comparisons.

Analyses were conducted using robust regression, adjusted for age and allelic class for combined data (“All” = FM only with incomplete silencing + PM/FM mosaic), while adjusted for age only in each of allelic subgroup; *FM* full mutation, *PM* premutation, *β* estimated regression coefficient, *se* standard error, *ABC* aberrant behavior checklist, *UI* utility index.

*p-value (*p*) remained < 0.05 after adjusting for multiple comparison using Bonferroni correction method.

## Discussion

This study for the first time reports intergroup comparisons of the behavioural phenotype, as measured by the ABC-C_FX_, between FXS males stratified based on the presence or absence of *FMR1* mRNA and CGG size mosaicism. It also reports novel associations between *FMR1* mRNA and ABC-C_FX_ scores in FXS. One of the key findings of this study is that stratification of the FM-only incomplete *FMR1* silencing and PM/FM mosaic groups revealed significant associations between mRNA levels and Irritability scores, ABC-C_FX_ total scores, and the UI, while no significant associations were observed when these two groups were combined. The study found elevated *FMR1* mRNA levels were associated with more severe irritability symptoms and maladaptive behaviours generally (ABC-C_FX_ total scores) and lower parent reported health-related quality of life (ABC-C_FX_ UI) in males with incompletely silenced FM alleles more specifically.

The findings in the incomplete silencing group suggest reactivation of large expanded alleles may have a toxic gain of function, particularly in terms of irritability. Taken together with our previous findings^11^, it is evident that residual mRNA from transcribed FM alleles has negative implications for behavioural outcomes in males with FM-only alleles. It is also possible that this may be explained by some of the individuals in the incomplete silencing group, harbouring a portion of PM or unmethylated FM alleles that are expressed and may be toxic in some cells. Further studies in larger, independent cohorts would assist in furthering our understanding of the impact of residual *FMR1* mRNA on the FXS behavioural phenotype. The findings also highlight that combining PM/FM mosaic and FM-only males with incomplete silencing may ‘wash out’ any relationships observed between *FMR1* mRNA levels and ABC-C_FX_-related clinical data, and also any effect of medications used in clinical trials.

This has been exemplified in the randomised placebo controlled trial of mavoglurant**,** an mGluR5 antagonist. Interestingly, this earlier study demonstrated no significant effects between mavoglurant and placebo from baseline to follow up on ABC-C_FX_ total scores in 30 males (18–36 years) with FXS^31^. However, when those individuals with a fully methylated promoter and *FMR1* mRNA silencing (*n* = 7) were analysed separately as a sub-group, significant improvements were seen from baseline to day 19 or 20 of treatment on ABC-C_FX_ total scores, for all these patients. In examining those with partial methylation, some individuals showed improvement and others demonstrated a worsening of maladaptive behaviours. The authors theorised that the variation in treatment response among those with partial methylation may be explained by the variation in *FMR1* mRNA and FMRP expression and that dosage may need to be reduced in these cases. Nonetheless, Berry-Kravis et al.^31^, reported the results of two randomised, double-blind, placebo-controlled trials on over 300 FXS patients using the same stratification method, and found no improvements in either group.

Of note in all these clinical trials participant inclusion criteria was a diagnosis of FXS described as either “confirmed by genetic testing”^31^, without further specification of the assays used and the CGG allelic class results, or simply defined as the presence of > 200 CGG repeats or a positive cytogenetic test accompanied by family history of FXS^32^. Therefore these trials may have included individuals with CGG size mosaicism. Jacquemont and colleagues^32^ noted that two FM males who were found to have *FMR1* mRNA levels within the control range may have in fact been PM/FM mosaic, but CGG sizing was not performed to confirm this. However, this assumption may only be partially true. As shown in Fig. 1, the *FMR1* mRNA levels in our cohort of males with PM/FM mosaicism partially overlapped with both the control and incomplete silencing FM-only groups. Therefore, there may have been more than two males with undetected PM/FM mosaicism in the Jacquemont cohort. Thus, results in these clinical trials may have differed if participants were stratified based on *FMR1* mRNA levels *and* presence or absence of PM/FM mosaicism.

Although it was expected that males with incomplete *FMR1* mRNA silencing would have significantly elevated scores on the ABC-C_FX_ compared to those males with complete *FMR1* mRNA silencing, this was not confirmed. This hypothesis was based on our previous findings demonstrating elevated ASD features, specifically more social affect difficulties, based on the Autism Diagnostic Observation Schedule-2nd edition (ADOS-2) in the incomplete silencing group^11^. However, no statistically significant differences were observed between the two FM-only groups. Instead the results demonstrated that the FM-only complete silencing group had significantly elevated scores on the Irritability and Inappropriate Speech subscales and ABC-C_FX_ total score compared to the PM/FM mosaic group. Additionally, the complete silencing group had a significantly lower ABC-C_FX_ UI, indicating poorer parent reported health-related quality of life. The incomplete *FMR1* silencing group also had significantly higher scores on the Inappropriate Speech subscale compared to the PM/FM mosaic group. Several factors may have contributed to the differences observed between this study and our previous study.

While both assessments relate to behavioural features that are commonly attributed to FXS, the ADOS-2 is undertaken by a trained clinician while the ABC-C is completed by the parent/caregiver. ADOS-2 assessors are required to undertake specialist training to identify autistic behaviours, whereas the ABC-C is completed by a parent/caregiver who may be less cognisant of the types and severity of these behaviours. Moreover, parent-report measures, such as the ABC-C, may be biased by the prognostic information that is given at the time of diagnosis. In a larger sample of FXS males from which this cohort was drawn, no significant differences were found on ADOS-2 scores between FM-only and PM/FM mosaic males^11^ and with the current sample the PM/FM mosaic group did not significantly differ to the two FM-only groups on ADOS calibrated severity scores (CSS), where ADOS-2 assessors were blinded to the allelic classification of the person being assessed. Nevertheless, the discrepancies in group differences in behavioural findings could also be underpinned by weak associations between ABC-C_FX_ scores and ADOS-2 CSS. Although both measures target overall similar behavioural problems, the ABC-C_FX_ subscales encompass some maladaptive behaviours which are not fully captured by the ADOS-2 CSS and vice versa.

The use of the ABC-C_FX_ may also contribute towards a lack of demonstrable efficacy in clinical trials. There are likely issues with biases based on the prognostic information parents are given about their child. It is plausible that the lower baseline scores on the ABC-C_FX_ for PM/FM mosaic males, as seen in the current study, may reduce the ability to observe clinically significant changes post treatment and may reduce effect sizes when combined with FM-only males, particularly if differences in ABC-C_FX_ scores at baseline are not accounted for in statistical analyses.

Another issue is the use of an ABC-C total score. The original developers of this measure highlight that a total score was never recommended, with explicit instructions in the manual stating “it is inappropriate to compute a total aberrant score based on summation of all 58 items, as the subscales are largely independent”^33^. Thus, compilation of a total score represents no specific construct^34^. While in the current study a significant association was observed between *FMR1* mRNA and the total score in the incomplete silencing group this is predominantly driven by the Irritability subscale. The Irritability subscale of the ABC-C_FX_ has been classified in the moderate to strong category of outcome measures for FXS and is increasingly used in clinical trials^35^. However, this subscale itself was shown to encompass four latent factors including tantrums, self-harm, verbal outbursts, and negative affect in a large sample of adolescents with idiopathic ASD^36^.

While parent-reports have their utility, development of objective outcome measures that complement and extend on parent reports are required. Furthermore, rather than using adapted versions of measures that were generated for general ID or other neurodevelopmental disorders, establishing FXS specific parent and clinician-report measures would be more appropriate. While such processes are time consuming, this may ultimately lead to more sensitive measures for FXS clinical trials. Moreover, development of objective assessments that can be administered repeatedly without ‘learned’ effects would be advantageous.

### Limitations

One of the main limitations of the current study is the inclusion of participants who were taking a psychoactive medication which may have impacted parent reports of behaviour. Intended and side effects of specific medications may also impact the specific behaviours that are reported on in the ABC-C^37^. However, the proportion of participants taking a psychoactive medication did not significantly differ between the three groups, and this same limitation is also applicable to most previous FXS studies. Future studies will explore relationships of ABC-C_FX_ with other molecular variables including FMRP, *ASFMR1* and *FMR1* promoter methylation, to further explain heterogeneity in the phenotypes and underlying biological mechanisms in different sub-groups of FXS.

Another limitation in the current study is the use of peripheral blood to analyse *FMR1* mRNA levels. While peripheral tissues, such as blood, are a relatively non-invasive way to examine gene expression, such tissues may not be entirely reflective of gene expression in the brain. Nonetheless, the findings reported here, as well as our previous findings demonstrating associations between *FMR1* mRNA in PBMCs with autistic features^11^ and *FMR1* methylation in buccal epithelial cells and intellectual functioning in males with FXS^14^, highlight the utility of using peripheral tissues to examine genotype–phenotype relationships.

## Conclusions

Despite advances in the understanding of the molecular underpinnings of FXS, clinical trials are yet to demonstrate efficacy in humans. As technological advances are being made, the understanding of the biology of FXS becomes more complex, with the likelihood that many sub-groups of FXS exist and will emerge. This study highlights how different sub-groups (FM-only with complete *FMR1* silencing, FM-only with incomplete *FMR1* silencing, and PM/FM mosaics) demonstrate different associations and intergroup differences between molecular and clinical outcomes. Although associations could not be undertaken for the complete silencing group (all *FMR1* mRNA values = 0), a large degree of variability was still seen on ABC-C_FX_ scores, suggesting that factors other than loss of *FMR1* mRNA are contributing to the phenotype heterogeneity and/or that molecular analyses in blood do not always reflect molecular changes observed in the brain and other tissues. Differences observed between FM only and PM/FM mosaic males on the ABC-C_FX_, in addition to the lack of associations with *FMR1* mRNA levels and ABC-C_FX_ scores in the PM/FM mosaic group highlight that patient stratification by presence or absence of PM/FM size mosaicism *and/or* incomplete silencing of FM allele mRNA may be valuable for participant stratification in future research and clinical trials.
